# Adoptive Cell Therapy for T-Cell Malignancies

**DOI:** 10.3390/cancers15010094

**Published:** 2022-12-23

**Authors:** Karen Kai-Lin Fang, Jong Bok Lee, Li Zhang

**Affiliations:** 1 Toronto General Hospital Research Institute, University Health Network, Toronto, ON M5G 1L7, Canada; 2 Department of Laboratory Medicine and Pathobiology, University of Toronto, Toronto, ON M5S 1A8, Canada; 3 Department of Immunology, University of Toronto, Toronto, ON M5S 1A8, Canada

**Keywords:** T-cell malignancies, adoptive cell therapy, CAR-T cell, NK cell, γδ T cell, DNT cell

## Abstract

**Simple Summary:**

Patients with relapsed and refractory T-cell malignancies have poor outlook and limited treatment options. Recently, adoptive cell therapy has emerged as a promising therapy for patients with T-cell malignancies. In this review, we examine the current progress on adoptive cell therapy for T-cell malignancies and discuss the potential future directions.

**Abstract:**

T-cell malignancies are often aggressive and associated with poor prognoses. Adoptive cell therapy has recently shown promise as a new line of therapy for patients with hematological malignancies. However, there are currently challenges in applying adoptive cell therapy to T-cell malignancies. Various approaches have been examined in preclinical and clinical studies to overcome these obstacles. This review aims to provide an overview of the recent progress on adoptive cell therapy for T-cell malignancies. The benefits and drawbacks of different types of adoptive cell therapy are discussed. The potential advantages and current applications of innate immune cell-based adoptive cell therapy for T cell malignancies are emphasized.

## 1. T-Cell Malignancies

T-cell malignancies are a heterogenous group of hematological cancers originating from dysfunctional precursor and mature T cells. T-cell malignancies can be broadly categorized into T-cell leukemias and T-cell lymphomas, with various subtypes based on the developmental stage and the location of T cells. T-cell malignancies are often aggressive and associated with poor prognoses. Among T-cell malignancies, more aggressive forms include T-cell acute lymphoblastic leukemia (T-ALL) and peripheral T-cell lymphoma (PTCL). T-ALL is a neoplasm of lymphoblasts committed to the T cell lineage and represents 15 to 25% of pediatric and adult ALL. PTCL affects mature T cells and accounts for 10 to 15% of non-Hodgkin’s lymphoma (NHL). 

The current first-line treatment for T-ALL and PTCL involves intensive multiagent combination chemotherapy. However, the overall survival of adult patients with T-ALL is less than 50% due to treatment-related toxicities and disease relapse [1,2], while more than 60% of patients with PTCL experience relapsed and refractory (r/r) disease with limited treatment options [3]. Allogeneic stem cell transplantation (HSCT) is a potentially curative therapy for patients at high risk of r/r disease. Still, allogeneic HSCT is limited by patient eligibility, availability of a matched donor, and significant risks of infections, graft-versus-host-disease (GvHD), and long-term complications. Altogether, the limited therapeutic options and a dismal outlook for patients with T-cell malignancies underscore the urgent need for a new line of therapy.

## 2. Adoptive Cell Therapy

**Figure 1:**
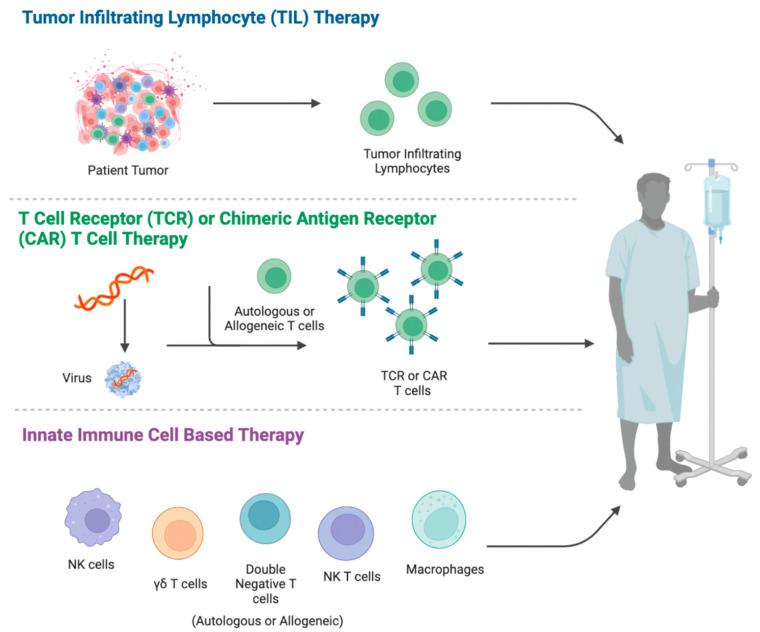
Types of adoptive cell therapy.

Immunotherapy has been coined as the “fourth pillar” of cancer treatment, an addition to the traditional three pillars of surgery, radiation, and chemotherapy. Immunotherapy is an emerging branch of medicine that modifies the host immune system or utilizes components of the immune system to treat cancer. In particular, adoptive cell therapy (ACT) has gained interest as an immunotherapy and achieved promising clinical results for hematological cancers, particularly for B-cell malignancies. ACT involves the infusion of ex vivo expanded immune cells into patients for treating cancers, ranging from re-infusing immune cells taken from specific tumor sites to infusing genetically modified donor-derived immune cells. Currently, four major types of cells are used in ACT: tumor-infiltrating lymphocytes (TILs), transgenic T cell receptor (tgTCR)-modified T cells (tgTCR-T cells), chimeric antigen receptor (CAR)-modified T cells (CAR-T cells), and unmodified or modified innate immune cells (Figure 1). 

One form of ACT involves the use of TILs. TILs comprise intratumor lymphocytes that can recognize and attack tumor cells. The procedure of TIL-based ACT was first developed for melanoma patients in the 1980s [4]. In TIL-based ACT, naturally infiltrating lymphocytes are isolated from the resected tumor materials and ex vivo expanded in the presence of a high dose interleukin-2 (IL-2) to therapeutic numbers, or approximately 10-100 billion cells. These cells are then infused into patients with high-dose IL-2 to support the growth and survival of infused cells in the tumor microenvironment. On average, TIL therapy has shown a response rate of 50% in patients with melanoma [5] and has since been examined for use in other solid tumors, including cervical cancer, breast cancer, colorectal cancer, and non-small cell lung cancer [6]. There are certain limitations to TIL therapy, including the exhaustion and dysfunction of TILs due to chronic stimulation during expansion and immunosuppressive tumor microenvironment and the limited applicability in hematological cancers. Nonetheless, the technical ability to isolate and rapidly expand T cells derived from patients and impressive clinical outcomes in patients without alternative treatment options has spearheaded the field of ACT. 

The second type of ACT involves tgTCRs to improve tumor recognition and the function of lymphocytes. Early studies showed that antigen specificity of T cells could be modified via tgTCR gene transfer [7]. T cells modified with virus-specific tgTCRs were shown to mediate antiviral activity [8] and promote the eradication of tumors expressing the viral epitope [9], while tgTCR-T cells specific for tumor-associated antigens (TAA) were shown to confer antitumor reactivity [10]. Since then, tgTCRs targeting numerous tumor- or virus-associated/specific antigens have been investigated for immunotherapy [11]. In the tgTCR-based ACT, tgTCRs have specificities for TAAs including overexpressed proteins, lineage-restricted proteins, and neoantigens derived from malignancy-restricted proteins. Relevant TAA-targeting tgTCRs are identified by investigating the TCR repertoires of TILs or peripheral blood T cells from patients. This allows engineered tgTCR-T cells to target TAAs found in solid and hematological malignancies. The potential efficacy of tgTCR-T cells has been examined in preclinical studies and clinical trials for hematological cancers such as acute myeloid leukemia (AML), myelodysplastic syndromes (MDS), B-ALL, and B cell lymphoma [12]. In addition, tgTCR-T cell therapy targeting minor histocompatibility antigens, such as HA-1, has been evaluated for preventing relapse after allogeneic HSCT in cases with donor-recipient mismatch [13]. However, only 16% of tgTCR-T cell-based clinical trials to date have aimed at treating hematological malignancies [14], and no clinical studies conducted to use tgTCR-T cell therapy for the treatment of T-cell malignancies. tgTCRs recognize antigens presented by MHCs, which allows it to target antigens from both intracellular and cell surface proteins. However, this makes tgTCR-T cell therapy MHC-restricted, a major limitation of this ACT. 

A more popular approach of ACT utilizes CAR-T cells. CARs are artificial receptors that are generated by combining the signaling domain of a TCR with a single chain variable fragment (scFv) specific for a target antigen. Unlike TCRs, CARs can redirect the cytotoxicity of T cells against target antigens independent of MHC, albeit generally against surface-expressed antigens. In contrast with TIL, CAR-T cell therapy has primarily been evaluated for use in hematological cancers. Notably, CAR-T cell therapy has achieved significant clinical success against B-cell malignancies, and the first CAR-T cell therapy was approved in 2017 for CD19-directed CAR-T cell therapy against r/r B-ALL and diffuse large B cell lymphoma (DLBCL). In a clinical study of CD19 CAR-T cell therapy for DLBCL, 58% of patients showed complete response, and 25% of patients showed partial response [15], highlighting the potent efficacy of CAR-T cell therapy. Recently, B cell maturation antigen (BCMA)-directed CAR-T cell therapy has also been approved for use in r/r multiple myeloma. CAR-T cell therapy is currently being investigated for a diverse range of solid and hematological tumors. Of note, there are growing numbers of preclinical and clinical studies focusing on CAR-T cell therapy for T-cell malignancies. Many of these studies demonstrated both the challenges and the promises of CAR-T cell therapy for T-cell malignancies, which will be highlighted in the next section.

While ACT conventionally utilizes CD4^+^ and CD8^+^ T lymphocytes for treating cancers, studies have also recently gained interest in developing ACT using other immune cell types. Innate immune cells, such as natural killer (NK) cells, gamma delta (γδ) T cells, and double negative (DN) T cells, are currently being investigated for use in ACT against several hematological malignancies, including AML, B-cell malignancies, and T-cell malignancies. Each of these immune cells has shown unique advantages, limitations, and suitability for ACT due to their distinct characteristics and functions in the immune system. While the use of innate immune cell-based ACT for T-cell malignancies is still in its infancy, there have been some encouraging results.

## 3. Targeting T-Cell Malignancy by CAR-T Therapy

**Figure 2:**
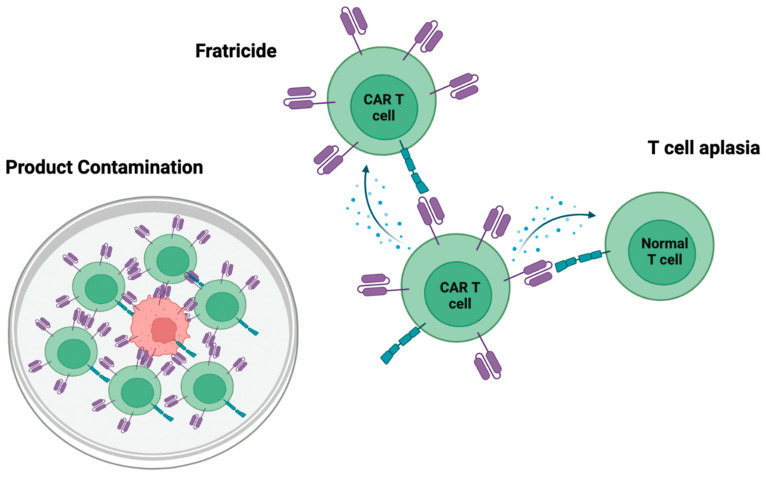
Challenges of using CAR-T cell therapy for T-cell malignancies.

The success of CAR-T cell therapy for B-cell malignancies has paved the way for the development of CAR-T cell therapy for T-cell malignancies. The potential of using CAR-T cells to target T-cell malignancies have been demonstrated in a number of preclinical studies, which have been extensively reviewed recently [16,17,18,19,20]. However, the application of CAR-T cell therapy for T-cell malignancies is more challenging due to the similarities between malignant T cells and effector CAR-T cells. The challenges of using CAR-T cell therapy for T-cell malignancies include the fratricide of CAR-T cells, CAR-T cell product contamination, and T-cell aplasia, as depicted in Figure 2. These challenges and potential solutions will be discussed in more details below.

### 3.1. Challenges

The fratricide or self-killing of CAR-T cells is a major obstacle encountered for the development of CAR-T cell therapy for T-cell malignancies. The use of CAR-T cell therapy necessitates the selection of targetable antigens present on tumor cells. Ideally, these antigens should be lineage-restricted and commonly found on tumors, but not expressed on hematopoietic stem cells to reduce toxic side effects. B-lineage-specific antigens, such as CD19, CD20, and CD22, are typically selected as targets for CARs to treat B-cell malignancies. For T-cell malignancies, potentially targetable antigens include pan T-lineage antigens, such as CD3, CD4, CD5 and CD7. These antigens are highly expressed in T-cell malignancies [17] but are absent in hematopoietic stem cells. However, since these T-lineage antigens are present on both malignant T cells and normal T cells, CAR-T cells directed to target these T-cell lineage antigens on blasts will inevitably target themselves. This leads to the fratricide of CAR-T cells during production and creates challenges in acquiring sufficient quantity of products for clinical use. 

The second challenge lies in the potential contamination of autologous CAR-T cell products. All FDA-approved CAR-T cell therapy uses autologous T cell from the peripheral blood of patients. In a case study, it was observed that the transduction of a single leukemic B cell with anti-CD19-CAR led to the masking of CD19 antigen on the leukemic cell and subsequent antigen-positive disease relapse [21]. In patients with T-cell malignancies, the T cell compartment is abnormal, and malignant T cells can often be found in the peripheral blood. As a result, a complicated and thorough process is needed to isolate only the normal autologous T cells from the patient blood for autologous CAR-T cell product manufacturing. Based on the immunophenotypes of malignant T cells in the enrolled subjects, clinical studies have used selection and sorting strategies to exclude malignant T cells. For patients with CD3^−^ blasts, only CD3^+^ cells were selected for CAR-T cell production [22], while for patients with abnormally high CD4^−^CD8^−^ T cells, only CD4^+^ and CD8^+^ T cells were used [23]. The final CAR-T cell products were then assessed by flow cytometry and/or qPCR to ensure there were no residual malignant T cells within the detection sensitivity range [22,23]. Nonetheless, such strategies are only applicable to certain cases and malignant T cells may also persist in the final CAR-T cell products at low levels. To avoid this challenge, the use of allogeneic CAR-T cells derived from healthy donors for therapy has been studied. However, donor-derived T cells pose the risks of graft-versus-host-disease (GvHD) and host rejection of infused allogeneic CAR-T cells leading to treatment failure. 

T-cell aplasia is the third obstacle. T cells play an important role in the immune system and are essential in fighting off infections and cancer development. The use of CAR-T cells that target T-lineage antigens would eliminate both malignant and normal host T cells, which may result in T-cell aplasia. While B-cell aplasia is also seen from CD19-CAR-T cell therapy, it is well tolerated in the clinic after immunoglobulin replacement treatment. In contrast, the effect of T-cell aplasia is not well studied and may result in AIDS-like immunodeficiency. Currently, there is no effective treatment for T-cell aplasia.

### 3.2. Solutions

Recently, studies have employed different strategies to overcome these challenges. These strategies predominantly involve additional genetic modifications of CAR-T cells but can also include other methods. The currently published preclinical approaches are summarized in Table 1. 

To avoid the fratricide of CAR-T cells, the best strategy is to select target antigens expressed only on tumors or shared with restricted T cell subsets. For example, CD37 is absent on T cells but found in some cases of PTCL [35], which makes it an ideal target for CARs. Similarly, CD1a expression is restricted to developing cortical thymocytes and found in some cases of cortical T-ALL [36,37], and CD30 expression is limited to a subset of activated lymphocytes in follicular regions of lymphoid tissues and found in some PTCLs [38]. The use of these antigens can limit fratricide since they are absent in circulating T cells [27,28,29]. However, such antigens are not expressed in the majority of T-cell malignancies, which limits the applicability of such a strategy. A more commonly taken approach to avoid fratricide is by abrogating the expression of the target antigen on CAR-T cells. For instance, to generate CD3^−^ or CD7^−^ specific CAR-T cells, studies have used gene editing tools such as TALEN and CRISPR/Cas9 [24,30] or a protein expression blocker [25] to abrogate CD3 and CD7 expression on T cells prior to CAR transduction. The absence of target antigen expression on CAR-T cells can mitigate fratricide. In addition, fratricide can be prevented by limiting CAR expression in vitro and allowing CAR to be expressed on T cells only after infusion to the host. For example, a study has implemented an inducible Tet-off system where CAR expression for CD5-specific CAR-T cells is regulated by a small molecule inhibitor, doxycycline [26]. In this design, CAR transcription is driven by a transactivator, and the binding of the transactivator to the CAR promoter is inhibited by doxycycline. Supplementation with doxycycline during ex vivo expansion of transduced T cells was shown to result in minimal CAR expression on the cell surface and mitigate the occurrence of fratricide. More recently, a study has also utilized pharmacologic inhibitors of the CAR/CD3ζ signaling kinases to prevent CAR-mediated fratricide [39]. Unedited CD7-CAR-T cells were shown to expand ex vivo without fratricide when supplemented with the inhibitors, ibrutinib, and dasatinib. Upon removal of these inhibitors, the cytotoxicity of these CD7-CAR-T cells was retained.

As previously mentioned, using allogeneic T cells is a strategy to avoid autologous CAR-T cell product contamination. This approach necessitates further modifications to allogeneic T cells to overcome the issues of GvHD and allorejection. Acute GvHD is commonly mediated by alloreactive donor TCRs. To avoid GvHD, studies have knocked out the TCRα chain and TCRαβ from T cells using CRISPR/Cas9 and TALEN-mediated gene editing [30]. These studies show that the TCRα gene knockout can prevent severe GvHD. However, achieving 100% knockout is not easy and studies have shown that leaky TCR knockout can lead to GvHD [40,41]. As an alternative approach, studies have examined using non-alloreactive T cells, including virus-specific T cells, to avoid GvHD. In a phase I clinical trial, CD19-CAR generated using virus-specific T cells were well tolerated in patients with B-cell malignancies who experienced relapse after allogeneic HSCT [42]. Nonetheless, the use of virus-specific T cells might not be completely safe due to alloreactivity [43,44] and incidences of GvHD following virus-specific T cell infusion have been observed in clinical trials [45]. Due to the low number of circulating virus-specific T cells, there is also difficulty acquiring sufficient number of cells for therapy [46,47]. Rejection of infused donor cells is another limitation associated with allogeneic CAR-T cell therapy, which hinders the therapeutic efficacy and the durability of the response. Allorejection is mediated by host T cell and NK cell recognition of donor MHC class I and II molecules. To avoid allorejection, studies have knocked out β2 microglobulin (β2M) and class II major histocompatibility complex transactivator (CIITA) from CAR-T cells to abrogate the expression of MHC class I and II molecules from CAR-T cells [48,49]. In addition, the expression of certain MHC class I molecules, such as HLA-E [50] or HLA-G [51], has also been suggested as a strategy to prevent NK cell-mediated allorejection. However, these approaches require extensive genetic modifications, which can lead to higher risk of genetic abnormality and raise the bar for quality control. Another strategy used by Allogene to avoid allorejection is host immune suppression. Studies have used anti-CD52 monoclonal antibody such as alemtuzumab during patient preconditioning alongside fludarabine and cyclophosphamide. Preconditioning depletes host lymphocytes and negatively selects CD52^+^ alloreactive T cells. Subsequently, patients are infused with allogeneic CAR-T cell products that are knocked out of CD52, which allows the CAR-T cells to be resistant to anti-CD52 antibody treatment [52]. However, such strong lymphodepleting preconditioning regimens can lead to severe and prolonged lymphopenia in recipients, which can render patients highly susceptible to infections. Alternatively, a study has armored CAR-T cells with an “alloimmune defense receptor” that selectively recognizes 4-1BB, which allows CAR-T cells to eliminate activated T cells [53]. Yet, this strategy can cause on-target toxicities to myeloid cells or other non-alloreactive cells that express 4-1BB and lead to more exhaustion due to constant CAR-T cell activation by different target cells. 

Unlike B-cell aplasia, T-cell aplasia can lead to life-threatening consequences. One strategy to mitigate T-cell aplasia is to target antigens expressed only on specific T-cell subsets to avoid completely eradicating T cells. For example, since TRBC1 and TRBC2 expression on T cells are mutually exclusive, a study has demonstrated that TRBC1-specific CAR-T cells can target TRBC1^+^ T-cell malignancies while sparing TRBC2^+^ T cells [34]. However, this approach only applies in certain cases where non-pan T-lineage antigens can be identified. In patients with T cell malignancies, TCR diversity is skewed and there is an abnormally high frequency of certain complementarity-determining region 3 (CDR3) sequences [54]. To limit pan T-cell aplasia and off-tumor toxicity, a study has proposed targeting of CDR3 epitope on TCRs of malignant T cells using CAR-T cells [33]. Since the TCRs and CDR3 epitopes on T cell germlines are unique for each patient, dominant CDR3 epitopes are identified via next-generation sequencing to generate personalized tumor-specific CAR-T cells. However, this approach can render the manufacturing process more expensive and time-consuming. Further, some propose to use CAR-T cells as a bridge therapy to allogeneic HSCT, while others utilize a suicide gene or safety switch, where a drug such as CAMPATH can be administered to eliminate persisting CAR-T cells once the desired therapeutic outcome is observed [32]. However, these approaches come at the expense of effector CAR-T cell persistence, which is paramount in achieving long-term therapeutic efficacy in cell-based therapies.

Overall, the preclinical studies have demonstrated the feasibility of genetically modifying CAR-T cells and showed the requirement for additional processes to mitigate challenges associated with targeting T-cell malignancies.

### 3.3. Clinical Trials

To date, there are at least four completed phase I clinical trials, two terminated phase I clinical trials, and 53 ongoing phase I and phase II clinical trials for T-cell malignancies using CAR-T cells, 35 of which are at the recruitment stage. These 59 studies focus on the use of CD4 (5 studies), CD5 (5 studies), CD7 (28 studies), CD30 (17 studies), CD37 (1 study), CD123 (1 study), CD147 (1 study), or TRBC1 (2 studies)-specific CARs to target a diverse range of T-cell malignancies, including T-ALL, PTCL, T cell lymphoblastic lymphoma (T-LBL), anaplastic large cell lymphoma (ALCL), adult T cell leukemia/lymphoma, angioimmunoblastic T cell lymphoma (AITL), enteropathy-associated T cell lymphoma, and extramedullary NK/T cell lymphoma. These clinical trials primarily investigate autologous or HSCT-donor-derived CAR-T cells (41 studies, 69%), healthy donor-derived allogeneic CAR-T cells (10 studies, 17%), or both (4 studies, 7%) for patients with r/r disease after chemotherapy with or without prior HSCT. A few trials evaluate HSCT-donor derived CAR-T cell therapy for patients post-HSCT to prevent disease relapse. Among CD7-specific CAR-T cells, various techniques are utilized to prevent fratricide, including CD7 expression blockade and genetic abrogation. For most studies on allogeneic donor-derived therapy, “universal CAR-T cells” are manufactured with TCRα subunit constant (TRAC) disruption to avoid GvHD, while virus-specific T cells are used in one study to navigate the issue of GvHD. Aside from active clinical trials, there is also a follow-up study examining the long-term effects of CD7-specific CAR (NCT05509855). All completed and ongoing clinical trials on CAR-T cell therapy for T-cell malignancies are listed in Table 2.

Sixteen of the completed and ongoing phase I clinical trials have published full manuscripts (3 studies) or preliminary results (13 studies), which are summarized in Table 3. These studies have investigated CD4^−^, CD5^−^, CD7, and TRBC1-specific CAR-T cells for r/r T cell leukemias and lymphomas and CD30-specific CAR-T cells for r/r T cell lymphomas. Most trials have achieved impressive results at one month follow-up. Of 13 trials where more than two patients (n = 3 to n = 22) were enrolled, 12 trials have reported complete remission (CR) rates that range from 66 to 100%, whereas in one trial the overall response rate (ORR) was 36.6% although 57% patients remained in stable disease (SD). However, trials with longer-term follow-ups showed that the responses achieved were not durable. For example, in one trial (NCT04572308), patients were treated with “naturally selected” CD7-specific CAR (NS7CAR) T cells [36]. NS7CAR-T cell products were generated by transducing bulk unmodified T cells with CD7 CAR. Hence, NS7CAR-T cell products are the cells that survive the fratricide during production. Following NS7CAR-T cell treatment, 19 out of 20 patients achieved CR in the bone marrow by day 28, and by two months, 14 out of 20 patients subsequently received HSCT, while four remained in CR. The limited durable response in this trial may be linked to the poorer viability and exhaustion of CAR-T cells following fratricide, suggesting the need to employ strategies to mitigate fratricide. In another trial (NCT04004637), patients were treated with “fratricide resistant” CD7-specific CAR-T cells, in which endogenous CD7 expression is blocked by tandem CD7 nanobody coupled with endoplasmic reticulum (ER)/Golgi retention motif [37]. Following treatment, 7 out of 8 patients achieved CR for three months, and two patients achieved CR for more than 12 months.

Despite the potential therapeutic efficacy in treating T-cell malignancies, these clinical trials also highlighted some limitations and risks involved in using CAR-T cell therapy. In all studies, patients experienced mild to moderate, and sometimes severe, adverse events. Of note, all patients in the two aforementioned studies experienced adverse events, where 19 out of 20 and 7 out of 8 patients experienced cytokine release syndrome (CRS) and two had neurotoxicities [22,23]. In other studies, adverse events commonly observed include grade I-III CRS, grade I-II immune effector cell-associated neurotoxicity syndrome (ICANS), and grade III-IV cytopenia. CAR target-specific T cell depletion has been expected and commonly observed. Some studies reported transient T-cell aplasia that was later recovered in 9 days to 3 months [22,59,61], while other studies noted a decrease in absolute T cell number but an increase in the number of normal T cells that lack target antigens [62,63,66]. In most studies, no unmanageable infections or infestations was observed; however, in rare cases, severe infections have occurred that resulted in death 2 to 6 months following treatment [65,66]. Less frequently, maculopapular rash and grade I-IV hematological toxicity have also been observed. In studies evaluating allogeneic therapy, incidences of mild GvHD have been observed [62,65,66]. In one trial (ChiCTR2000034762), 12 out of 20 patients experienced grades I-II GvHD following treatment with HSCT donor-derived or healthy donor-derived CD7 CAR-T cells for r/r T-ALL [66]. 

Furthermore, there are additional issues that may be involved in using CAR-T cell therapy for T-cell malignancies due to the need to avoid fratricide, product contamination with blasts and T-cell aplasia. For example, with the use of gene editing tools, there are risks of unwanted on-target and off-target effects, including pan-cytopenia and tumorigenesis. Such risks are only elevated with the increased complexity of gene modifications. Moreover, multiple gene modifications render the manufacturing process of CAR-T cells more complicated and costly. It is difficult, time-consuming, and laborious to generate and isolate T cells that have undergone multiple modifications with 100% or high enough purity. Preclinical studies have reported a poor expansion and yield for CAR-T cells following the knockout of CD3 and TCRαβ [30] and found that knocking out TCRs can reduce the persistence of CAR-T cells [70]. Altogether, these additional modifications can pose challenges such as lower efficacy, reduced yields, higher costs, and longer production times. Hence, while these studies have shown the potential efficacy of CAR-T cells, further improvements are needed to achieve a more durable response with minimal adverse events.

## 4. Targeting T-Cell Malignancies by Innate Immune Cells 

Due to the unsolved issues posed by using conventional CD4^+^ and CD8^+^ T-lymphocytes for T-cell malignancies, other immune cells are being investigated for use in ACT. The cell types primarily encompass innate immune cells, such as NK cells, γδ T cells, and DNTs. These immune cells occupy unique roles in the immune system and have distinct characteristics that can be leveraged to develop off-the-shelf allogeneic ACT. These innate immune cells are currently being studied as standalone and CAR-based therapies. The potential advantages and limitations of ACT using these immune cell types are summarized in Table 4 and will be discussed below. 

### 4.1. NK Cells

NK cells are innate immune cells defined by the expression of CD56 without CD3 or TCR. NK cells provide a first line of defense against malignant or virally infected cells. Unlike T cells, NK cells recognize malignant cells independent of antigen presentation and MHC restriction. Instead, NK cell activity is regulated by signals from an array of activating, inhibitory, and cytokine receptors that interact with specific ligands. Some important activating receptors include NK group 2D (NKG2D) and nature cytotoxicity receptors (NCRs), such as NK protein 30 (NKp30) and NK protein 44 (NKp44). These activating receptors bind to stress-induced molecules often expressed on malignant cells. To prevent self-recognition, NK cells express inhibitory receptors, such as killer cell immunoglobulin-like receptors (KIRs), which send inhibitory signals upon interactions with healthy self MHC-class I molecules. While tumors can often evade T cell recognition by downregulating MHC molecules, NK cells become activated when encountering cells that do not express self MHC molecules. Upon activation, NK cells produce rapid effector functions, releasing proinflammatory cytokines and cytotoxic granules to promote immune response and cause tumor cell lysis. Further, NK cells express CD16a, which can be activated by binding to antibodies deposited on malignant cells and induce antibody-dependent cell-mediated cytotoxicity (ADCC).

The antitumor effect of allogeneic NK cells was first demonstrated in HSCT for hematological malignancies. In T cell-depleted allogeneic HSCT, donor NK cells were the first immune cell population to recover and control residual malignant cells [71,72,73]. In HSCT with mismatched donor KIRs and recipient HLA class I molecules, NK cells were shown to have enhanced graft-versus-tumor (GvT) activity due to the absence of inhibitory signals [74]. Early studies of ALL, including pediatric T-ALL, showed that NK cells from KIR-mismatched HSCT reduced the incidence of disease relapse [71,75]. Further, a reduced number and function of NK cells were observed in T-ALL patients with active disease [76], providing a rationale for restoring NK cell function to improve patient outcomes. The intrinsic antitumor mechanism of KIRs and the aberrant NK cell activity in patients have provided the basis for developing allogeneic NK cell therapy. Adoptive transfer of NK cells has since been studied in various solid tumors and hematological malignancies, with or without HSCT [77]. In these studies, allogeneic NK cells have been shown to be safe with no GvHD-inducing activity, highlighting the potential of developing NK cells as an off-the-shelf product.

For T-cell malignancies, NK cell-based ACT have only been in evaluation for concurrent use with chemotherapy or post-HSCT. To date, there have been 5 clinical trials that investigated NK cell infusion for patients with r/r T-cell leukemia and lymphoma. Two of the 5 studies have been completed but have yet to report the outcomes. For the remaining 3 studies, one is in the recruitment stage, one has unknown status, and the other has been terminated due to low recruitment. Several trials have also investigated NK cell infusion post-HSCT for hematological malignancies but have not specified if any patients with T-cell malignancies are enrolled. The scarcity of clinical trials for T-cell malignancies may be related to early findings that donor NK cells in KIR-mismatched HSCT were beneficial for pediatric ALL but not adult ALL, which lacks adhesion molecules essential for NK cell activation. The clinical trials using NK cell therapy for T-cell malignancies are summarized in Table 5.

To utilize and improve the endogenous cytotoxicity of NK cells against tumors, studies have armoured NK cells with CARs. CAR-modified NK cell therapy is an emerging area of study, particularly in T-cell malignancies as NK cells do not share T-lineage antigens and are safe to be used in allogeneic settings. Since T and NK cells differ in hematopoietic lineage, fratricide can be avoided without modifying the surface antigen expression on NK cells. With these advantages, preclinical studies have used CAR-NK cells to target CD3^+^, CD4^+^, CD5^+^ and CD7^+^ T-cell malignancies. CAR-NK cells have shown potent antitumor activity against T-ALL and PTCL in vitro and in xenograft models [78,79,80,81,82,83]. Currently, there has not yet been any clinical trial of CAR-NK cells for T-cell malignancies. However, in clinical trials of CAR-NK cells for B-cell malignancies, CAR-NK cells have been found to have a favorable safety profile [84,85]. In one study of cord blood-derived CD19-CAR NK cells, 8 out of 11 patients responded to treatment with no observed CRS or neurotoxicity [84]. Further, no GvHD was observed despite HLA mismatch between patients and CAR-NK cell products. In addition, combining NK cell-based therapy with antibody treatment to utilize their ADCC mechanism has been suggested. The promising results from these studies suggest CAR NK cells’ unique potential for treating patients with T-cell malignancies.

However, there are also several challenges involved in using NK cells. NK cell expansion and persistence in recipients depend on cytokines such as IL-2 or IL-15, which are associated with toxicities when given systemically. In allogeneic NK cell therapy, there is a risk of immune rejection by host cells, especially in cases where systemic IL-15 supplementation is used to promote NK cell expansion [86]. Studies are currently investigating strategies similar to those employed for T cells, such as modifications to MHC class I or II molecules, to navigate the issue of immune rejection [87]. Another major issue in NK cell-based ACT is the source of NK cells [88]. NK cells constitute about 10% of lymphocytes in the peripheral blood. NK cells from the peripheral blood usually have mature phenotypes and are difficult to expand [89]. These NK cells are sensitive to extreme temperatures and show poor viability post cryopreservation, which may limit their feasibility as off-the-shelf therapy [90]. NK cell cultures also usually require feeder cells [91,92], increasing the risks of contamination and making the manufacturing process more costly and complicated. NK cells can also be derived from umbilical cord blood; however, cord blood-NK cells usually have a less mature phenotype with less potent cytotoxicity against tumors [93,94]. Alternatively, many studies used a lymphoma-derived NK cell line, NK-92. which is more standardized, easier to culture, and more manipulatable [95]. All preclinical studies on CAR-NK cells for T-cell malignancies have used NK-92 cells. NK-92 cells are currently being investigated in clinical trials for other types of malignancies [96]. Nonetheless, since NK-92 cells were originally derived from a lymphoma, they must be irradiated prior to use and thus have the issue of persistence [97]. Additionally, even with irradiation, the potential risks of tumorigenesis in patients are not complete resolved. Further, NK-92 cells are IL-2 dependent and require a repeated injection of IL-2 that may cause toxicities [98].

With the advances in induced pluripotent stem cells (iPSC), iPSC-derived NK cells have also been investigated as a potential source of NK cells [99]. iPSCs have unlimited proliferative capacity, and a single iPSC is sufficient to differentiate into a large number of NK cells for clinical use. iPSCs can be genetically modified prior to differentiation and produce a highly homogenous population. For example, studies have demonstrated that CAR-engineered iPSCs can differentiate into CAR-NK cells, which have shown potent antitumor activity in xenograft models [100,101]. To reduce the risk of alloreactivity, multiple gene modifications, including β2M and CIITA knock-out and HLA-E knock-in, have also been introduced to CAR-iPSC-NK cells [102]. Other modifications, such as IL-15 and safety switch, have also been introduced to further improve CAR-iPSC-NK cells [102]. Hence, iPSC-derived NK cells provide a potentially flexible avenue for generating a highly standardized and truly off-the-shelf product. However, iPSC products currently encounter several bottlenecks. While more robust protocols for iPSCs are being developed [103], the process of manufacturing iPSCs remains complex and lengthy. Further, iPSCs carry the risks of tumorigenicity, which has been shown in several animal studies where tumors developed from infused iPSCs [104]. Hence, the safety of iPSC products in human must be determined. 

Besides the issues encountered by NK cells, CAR-NK cells face additional challenges during the manufacturing process. NK cells have a lower transduction rate than conventional T cells, which may be due to the intrinsic resistance of NK cells against viral transduction [105]. Since CAR was originally designed for T cells with the intracellular domain of TCR, different CAR constructs and transduction protocols may be required to optimize the performance of CAR NK cells. While the short lifespan of NK cells has been considered to be advantageous by some studies to avoiding prolonged T-cell aplasia, the lack of persistence is still a challenge. Ex vivo expanded NK cells have an average lifespan of 14 days in patients. In a study of cord blood-derived CD19-CAR NK cells, the published results for circulating CAR NK cells detected by flow cytometry in complete responders were limited to the first 3 weeks [84,101]. In contrast, studies have shown that CD19-CAR-T cells were clearly detectable by flow cytometry for at least 6 months in most responders and have been detected even years after infusion [106,107,108]. Studies have shown that persistence in CAR-based therapy is essential for durable antitumor efficacy, and patients who received CAR NK cell therapy had to receive subsequent maintenance treatments to overcome the lack of long-term response of NK-cell based therapies [84]. CAR-NK cell therapy may require multiple infusions to achieve long-term efficacy, albeit at the cost of increased risks for immune rejection. Consequently, many NK-cell experts have focused on improving NK cells’ persistence and generating memory-like NK cells [109,110].

### 4.2. γδ T Cells

γδ T cells are a small subset of T lymphocytes that express a combination of a Vγ TCR chain paired with a Vδ chain. γδ T cells account for 1–10% of circulating T cells in the peripheral blood and more than 20% of intraepithelial T cells in the intestine [111,112]. Around 70% of γδ T cells are CD4^−^CD8^−^, and 30% are CD4^+^ or CD8^+^ [113]. γδ T cells are considered to bridge innate and adaptive immune responses, expressing clonally rearranged TCR genes yet capable of producing rapid effector response without prior sensitization [114,115]. Unlike conventional αβ T cells, γδ T cells typically recognize ligands independent of MHC restriction [116,117]. γδ T cells have multiple roles in the immune system, with both effector and regulatory functions [118,119]. Of interest, γδ T cells have been shown to have intrinsic cytotoxicity against malignant cells through different modalities [120,121,122,123]. γδ TCRs can be activated by endogenous tumor-derived phosphoantigens presented by butyrophilin molecules [124]. γδ T cells express several activating receptors shared with NK cells, such as NKG2D, NKp30 and NKp44 [125,126], which participate in malignant cell recognition. γδ T cells also express CD16a [127,128], which induces tumor cell death through ADCC, and FasL (CD95L) [129], which allows for the recognition of CD95 on tumor cells to initiate apoptosis. Upon activation, γδ T cells can release cytotoxic granules to lyse tumors and cytokines to amplify the immune response [130]. In addition, γδ T cells can act as antigen-presenting cells (APCs) to present tumour-derived peptides and activate the adaptive immune response [131,132]. 

In preclinical studies, γδ T cells have been shown to have antitumor effects against a broad spectrum of hematological and solid malignancies, including acute myeloid leukemia (AML) [133], B cell lymphoma [134], neuroblastoma [135], melanoma [136], and glioblastoma [137]. The presence of γδ T cells in TILs has been found to have the best correlation with favorable clinical outcomes compared to other immune cell types [138]. These results demonstrated the relevance of γδ T cells in cancer and provided a basis for the adoptive transfer of γδ T cells for cancer immunotherapy. Studies have particularly focused on developing γδ T cell-based ACT using Vγ9Vδ2 T cells, which comprise roughly 90% of γδ T cells in the peripheral blood. Since early studies demonstrated that aminobisphosphonates could activate Vγ9Vδ2 subset to mediate antitumor activity [139], there have been clinical trials using autologous ex vivo expanded Vγ9Vδ2 T cells for a diverse range of solid tumors [140]. As γδ T cells are MHC-independent, the risks for GvHD are low. Allogeneic Vγ9Vδ2 T cell-based ACT has also been evaluated in post-HSCT to control residual cancer or as a standalone therapy for advanced hematological malignancies [141,142]. In these trials, the clinical responses were often moderate and short-lived; however, Vγ9Vδ2 T cells were found to be well tolerated with no major adverse effects reported [141,142,143,144], which demonstrated the feasibility of Vγ9Vδ2 T cells as an off-the-shelf therapy. In addition, recent studies have explored the use of Vδ1 T cells for ACT. Unlike Vγ9Vδ2, Vδ1 T cells are primarily tissue-resident [112], and represent less than 1% of the peripheral blood mononuclear cells [145]. However, manufacturing protocols have recently been developed to allow for large-scale expansion of peripheral blood Vδ1 T cells to clinically relevant numbers [146,147]. Clinical-grade Vδ1 T cells have shown antileukemic effect against chronic lymphocytic leukemia and AML in preclinical studies [146,148], and a phase I clinical trial investigating allogeneic Vδ1 T cell-based ACT for AML was initiated (NCT05001451). To date, only one clinical trial has investigated γδ T cell infusion for T-cell malignancies. This trial focuses on allogeneic γδ T cell infusion for patients with r/r PTCL and is currently recruiting. There are also 3 other clinical trials, all of which are currently in recruitment, assessing γδ T cell infusion post-HSCT in patients with ALL. All trials are summarized in Table 6.

To improve the efficacy of γδ T cells, researchers have developed CAR γδ T cells. Studies have shown that γδ T cells can achieve similar levels of transduction efficiency as αβ T cells. In preclinical studies, CAR γδ T cells have been shown to be effective against various malignancies, including B-cell malignancies [149,150], neuroblastoma [151], melanoma [152], and hepatocellular carcinoma [153]. For T-cell malignancies, γδ T cells provides a unique opportunity to target αβ TCR^+^ T-cell malignancies without fratricide. αβ TCR is expressed on >95% of PTCL [154] and >30% of T-ALL [155]. A study has used anti-γδ CAR αβ T to target γδ T-cell malignancies [156], suggesting that a similar strategy can be employed for targeting αβ T-cell malignancies using anti-αβ CAR γδ T cells. In addition, CAR γδ T cells have been shown to retain antigen presenting ability [151], which may aid in triggering host adaptive immune response. While there is currently no clinical trial on CAR γδ T cells for T-cell malignancies, a handful of clinical trials have been initiated to evaluate allogeneic CAR γδ T cells for CD20^+^ B-cell malignancies and NKG2D^+^ solid tumors. In one trial of allogeneic off-the-shelf CD20-CAR γδ T cells for r/r B-cell lymphoma (NCT04735471) [157], 11 out of 16 patients achieved CR by day 28 and at least two patients remained in CR at 6 months follow-up. The treatment was found to be well tolerated, with no observed GvHD or grade III or above CRS or ICANS. With a safety profile and unique properties, CAR γδ T cell therapy for T-cell malignancies has gained recognition as a worthwhile area of study. 

Nonetheless, there are currently several limitations to using γδ T cell therapy for T-cell malignancies. Similar to NK cells, the short lifespan of γδ T cells is both an advantage for avoiding T-cell aplasia and a disadvantage for lacking persistence. Due to the short lifespan of γδ T cells [158], multiple doses may be required to attain the desired effect, and there is currently limited research on how to improve the persistence of γδ T cells to achieve long-term efficacy. While γδ T cells have not been shown to cause GvHD, there is limited research on the risk of immune rejection, which is a factor worth considering for developing γδ T cells as an allogeneic off-the-shelf product, especially when repeated injections are required. In addition, a study has reported that the freezing/thawing cycle significantly decreased the viability of Vδ2 γδ T cells [159]. As the ability to withstand cryopreservation is important for off-the-shelf products, more research is required to mitigate this effect. Finally, since γδ T cells express T-lineage antigens, fratricide is likely to occur when CAR γδ T cells are used to target most pan T-cell antigens. A study attempted to prevent fratricide by modifying γδ T cells with a CD5-specific CAR that lacks a signaling domain but allows for antigen-specific interaction with tumors and the subsequent tumor killing mediated by the endogenous antitumor mechanism of γδ T cells [160]. However, these CAR γδ T cells showed only moderately enhanced cytotoxicity against CD5^+^ T-ALL in vitro. This suggests that surface antigen modifications of γδ T cells are likely necessary to avoid fratricide while preserving higher antitumor efficacy. 

### 4.3. DNT Cells

DNTs are mature unconventional T cells defined by the expression of CD3 without CD4 or CD8 expression. Circulating DNTs comprise 3–5% of peripheral blood T lymphocytes, while tissue-resident DNTs have been observed in various organs, including the intestine, kidney, lung, liver, heart, genital tract, and central nervous system [161,162,163,164,165]. DNTs comprise both TCRαβ^+^ T cells and TCRγδ^+^ T cells, with proportions that vary between individuals. The origin of DNTs is currently not well understood. There are evidence supporting that peripheral DNTs originate from both thymic and non-thymic origin as thymectomized mice showed reduced, but detectable, levels of DNTs [166]. In the thymus, late-stage DN thymocytes can give rise to TCRαβ^+^ or TCRγδ^+^ DNTs, which possibly leave the thymus and expand in the periphery [167,168]. Thymus-independent pathways have also been described where activated peripheral CD4^+^ or CD8^+^ T cells can give rise to peripheral DNTs [169,170,171,172]. In the host immune system, both TCRαβ^+^ DNTs and TCRγδ^+^ DNTs have been shown to have effector and regulatory functions and are capable of recognizing target antigens without MHC restriction [173,174]. DNTs have been shown to infiltrate solid tumors [175,176]. A study showed that DN TILs in hepatocellular carcinoma consisted of activated TCRαβ^+^ DNTs and TCRγδ^+^ DNTs [177], suggesting the potential role of DNTs in cancer immunity. 

Of interest, our lab has shown that ex vivo expanded DNTs have antitumor effects against a variety of cancer types, including AML and non-small cell lung cancer [178,179]. DNTs express cytotoxic receptors, including NKG2D, DNAM1, TRAIL, and FasL, for the recognition and lysis of malignant cells [179]. Upon activation, DNTs can release high levels of IFNγ, TNFα, perforin, and granzymes [179]. Ex vivo expanded DNTs demonstrate potent cytotoxicity toward both autologous and allogeneic AML cells and are more cytotoxic toward allogeneic AML cells than ex vivo expanded CD8^+^ T cells [180]. Ex vivo expanded DNTs derived from healthy donors have potent cytotoxicity against chemotherapy-resistant AML [181], highlighting their potential as an allogeneic therapy for hematological malignancies. Importantly, DNTs have been shown to fulfill the requirements of an off-the-shelf therapy in our preclinical studies [182]. DNTs show the capacity to expand to therapeutic numbers and to maintain viability and antitumor function upon cryopreservation for at least 600 days [182]. Further, allogeneic DNTs are resistant to immune rejection [182,183], and αβ-DNTs can actively suppress GvHD [184]. These characteristics support DNTs as a candidate for an off-the-shelf product that does not require additional gene modifications. Recently, the safety of DNTs as an off-the-shelf ACT has been demonstrated in a phase I clinical trial, where infusion of allogeneic DNTs did not cause greater than grade II toxicities or any GVHD in AML patients that relapsed after allo-HSCT [185]. This study supports the off-the-shelf potential of allogeneic DNTs without the need for any genetic modifications. 

In addition, it was shown that the antitumor efficacy of DNTs can be improved by CAR transduction. In a recent report, CD19-CAR DNTs showed potent efficacy against B-cell malignancies, comparable to that of CD19-CAR conventional T cells while retaining its off-the-shelf characteristics [183]. The study demonstrated the potential of DNTs as a vehicle for CAR-based therapy. For targeting T-cell malignancies, DNTs have an important advantage as they lack of CD4 and CD8 expression. CD4 and CD8 are highly expressed in T-cell malignancies. A study with a cohort of 180 patients showed that 78% of T-ALL expressed CD4 and/or CD8 [186], while CD4 and/or CD8 is also highly expressed in PTCL, with varying degrees of expression depending on the subtype [187]. The high expression of CD4 and CD8 in T-cell malignancies and their absence on hematopoietic stem cells render CD4 and CD8 attractive targets for CAR-redirected cytotoxicity. Since DNTs express neither CD4 nor CD8, CAR DNTs can be redirected to target T-cell malignancies through these two T-lineage antigens without concerns of fratricide. 

Nonetheless, DNTs constitute a small percentage of the peripheral blood, and there is donor variability in the number DNTs that can be expanded. We have studied DNTs from about 200 healthy donors and observed that DNTs from majority of donors can be easily expanded ex vivo and possess potent antitumor activity. However, about 15% healthy donors’ DNTs show poor expansion rate and low cytotoxicity against tumor cell lines. Therefore, pre-screening of donors to exclude 15% of poor DNT-expanders is needed. Murine and human studies show that αβ-DNTs have potent immune-regulatory functions, where it can prevent GvHD and graft rejections [184,188,189,190,191,192,193,194]. This suggests that αβ-DNTs provides protections to infused DNTs from host rejection. However, their impact on the host immune system needs to be further investigated. Compared to other cell types, there is limited understanding of the biology of DNTs. Hence, more research is necessary to evaluate the functions and characteristics of DNTs and the feasibility of using DNTs for ACT.

## 5. Future Directions 

In addition to conventional T cells and innate immune cells, other emerging cell types may be suitable for use in ACT, such as NK T cells [195] and macrophages [196]. ACT using these cell types is being investigated in other tumors, including B-cell malignancies. While research on T-cell malignancies is currently trailing behind, it is anticipated that these cell types will be examined for use in T-cell malignancies in the near future. 

However, several challenges remain in using ACT for T-cell malignancies and other malignancies in general. There are currently two conflicting needs for T-cell malignancies: the need for T cell persistence to achieve long-term therapeutic efficacy and the need to avoid T-cell aplasia. It is thus paramount to find new tumor-restricted or subset-restricted targets to minimize the effect of T-cell aplasia. For the use of ACT, suppressive tumor microenvironments, antigen escape, and lack of cellular infiltration continue to be significant obstacles. As mentioned above, identification of antigens expressed on T-cell malignancies but not on normal T cells, appropriate uses of ACT as bridge therapy and suicide genes need to be further investigated. Further, studies are now steering towards using ACT in combination with other forms of immunotherapy, such as checkpoint inhibitors, oncolytic viruses [197], the stimulation of host dendritic cells [198], and adoptive transfer of dendritic cells [199] (NCT05277753). The applicability of the findings from these recent developments to the use of ACT in T-cell malignancies needs to be thoroughly considered.

## 6. Conclusions

ACT is emerging as a new line of therapy for patients with hematological malignancies. While there are currently challenges in applying ACT to T-cell malignancies, preclinical and clinical studies investigate various approaches to navigate these challenges. Although current ACT typically uses the autologous conventional T cells, different ACT types are being developed to harness the unique functions and properties of other immune cell types for allogeneic therapy. Innate immune cell-based therapies have demonstrated ample potential and garnered interest as a promising area of research for T-cell malignancies. 

## Figures and Tables

**Table 1:** Preclinical CAR-T cell approaches against T-cell malignancies.

Challenges	Solutions	Examples of CAR-T Cell Product	Modifications of CAR-T Cell	Limitations	Reference
Fratricide	CAR-T surface antigen modifications	CD7 CAR-T	CD7 knockout (KO)	Risks of unwanted effects from gene editing	[24]
			CD7 protein expression blocker	Requires multiple transductions, which may reduce yield	[25]
	Reversible CAR expression	CD5 CAR-T	Tet-off	Side effects from drug administration	[26]
	Use of antigens restricted to specific T cell subsets or tumors	CD1a CAR-T	None	Only applicable to some cases	[27]
		CD30 CAR-T			[28]
		CD37 CAR-T		Only applicable to some cases	[29]
	Use of alternative cell types	-	-	Depends on the cell type used	-
Product contamination	Use of allogeneic CAR-T	CD3 CAR-T	CD3 & TCRαβ KO	Requires TCR modification to avoid GvHD	[30]
		CD7 CAR-T	CD7 & TRAC KO		[31]
		-	-	Requires MHC modification to avoid rejection	-
	Use of alternative cell types	-	-	Depends on the cell type used	-
T-cell aplasia	Safety switch	CD4 CAR-T	Use of drug CAMPATH	Potential lack of long-term efficacy and cancer recurrence	[32]
	Use of antigens restricted to tumors	CDR3 CAR-T	None	More time-consuming and expensive	[33]
	Use of antigens restricted to specific T cell subsets	TRBC1 CAR-T	None	Only applicable to some cases	[34]
	Use of short-lived alternative cell types	-	-	Potential lack of long-term efficacy and cancer recurrence	-
	Bridge to HSCT	-	-	Only some patients are eligible Risks of complications and mortality involved in HSCT	-

**Table 2:** List of clinical trials of CAR-T cell therapy for T-cell malignancies.

Treatment Approach	Source	Cell Modifications (If Specified)	Phase	Study Stage	Actual or Estimated Enrollment	Clinical Trial Identifier
CD4 CAR-T	Autologous		I	Active	20	NCT03829540
			I	Terminated	9	NCT04973527
			I	Terminated	4	NCT04219319
				Active	50	NCT04712864
		IL-15 secretion	I	Active Preliminary results reported (n = 3)	12	NCT04162340
CD5 CAR-T	Autologous	CD5 KO via CRISPR/Cas9	Early I	Not yet recruiting	18	NCT04767308
	Autologous or HSCT donor		I	Active Preliminary results reported (n = 5)	42	NCT03081910
		IL-15 secretion	I	Active Preliminary results reported (n = 1)	20	NCT04594135
	HSCT donor or new donor		I	Recruiting	18	NCT05487495
			I	Active Preliminary results reported (n = 5)	18	NCT05032599
CD7 CAR-T	Autologous		I	Recruiting	15	NCT05554575
		CD7 blockade with ER anchor	I	Active Preliminary results reported (n = 5)	20	NCT04840875
			I	Recruiting	9	NCT04480788
			I	Recruiting	20	NCT05513612
			I	Recruiting	18	NCT05398614
		pharmacologic inhibitors ibrutinib and dasatinib in vitro	I	Recruiting	21	NCT03690011
			I/II	Recruiting	108	NCT04599556
			I	Recruiting	4	NCT05290155
		CD7 blockade with tandem CD7 nanobody	I/II	Recruiting	20	NCT04762485
			II	Recruiting	20	NCT05059912
		CD7 blockade with tandem CD7 nanobody	I	Completed Full manuscript published	8	NCT04004637
	Autologous or HSCT donor		I	Completed Full manuscript published	20	NCT04572308
	Autologous or donor		N/A	Active Preliminary results reported (n = 8)	100	NCT04916860
	Autologous or HLA-matched sibling donor	CD7 blocking	I	Active Preliminary results reported (n = 10)	24	NCT04823091
	HSCT donor or new donor	IntraBlock technology (CAR with ER retention of CD7)	I	Active	20	ChiCTR2000034762
			II	Recruiting	70	NCT04689659
	Allogeneic (healthy donor)	Universal CAR-T	Early I	Recruiting	30	NCT04264078
		Universal CAR-T	Early I	Not yet recruiting	15	NCT04860817
		Base edited via CRISPR/Cas9	I	Recruiting	10	NCT05397184
		TRAC, CD7 and HLA-II KO via CRISPR/Cas9	N/A	Recruiting	24	NCT04620655
		TRAC and CD7 deleted	I	Not yet recruiting	48	NCT05377827
		CJD7 and TRAC deleted	I/II	Recruiting	44	NCT04984356
			I	Recruiting	30	NCT05127135
	Unspecified	Non gene edited	I	Recruiting	24	NCT05212584
		Non gene edited	I	Recruiting	20	NCT04934774
		CD7protein expression blocker	I	Recruiting	20	NCT05043571
			N/A	Recruiting	100	NCT04928105
CD30 CAR-T	Autologous		I	Recruiting	50	NCT04008394
			I	Active	18	NCT02663297
			I	Completed Full manuscript published	9	NCT01316146
			I/II	Active Results reported for HL only	40	NCT02690545
			I	Active Results reported for HL only	66	NCT02917083
			I	Recruiting	20	NCT03383965
			I/IIa	Recruiting	30	NCT04653649
			I	Active	21	NCT04526834
		CCR4 receptor expressed	I	Active Preliminary results reported (n = 12) [55]	59	NCT03602157
			I	Completed Outcome reported	26	NCT03049449
			I	Active Preliminary results reported (n = 12) [56]	20	NCT04083495
			Early I	Not yet recruiting	9	NCT05208853
		CAR infused with iCasp9	I/II	Unknown Result reported for HL only [57]	20	NCT02274584
	Allogeneic (Healthy donor)	Use EBV-specific T cells	I	Active Preliminary results reported (n = 8) [58]	18	NCT04288726
			I	Not yet recruiting	18	NCT04952584
CD37 CAR-T	Autologous		I	Recruiting		NCT04136275
CD70 CAR-T	Allogeneic	TCR and MHC I KO via CRISPR/Cas9		Recruiting	45	NCT04502446
CD123 CAR-T	Autologous		I	Recruiting	32	NCT04318678
CD147 CAR-T	Autologous		Early I	Not yet recruiting	12	NCT05013372
TRBC1 CAR-T	Autologous		I/II	Active Preliminary results reported (n = 10)	200	NCT03590574
			I	Recruiting	9	NCT04828174
Multi-CAR-T	Autologous or HSCT donor		I/II	Recruiting	30	NCT04033302

**Table 3:** Results from clinical trials of CAR-T cell therapy for T-cell malignancies.

Treatment Approach	Manufacturing Method	Trial Stage, Status and Design	Number of Patients in Report	Age of Patients	Disease Type and Status	Disease Outcome	Treatment Associated Toxicities	Clinical Trial Identifier	Reference
CD4 CAR-T	- Autologous - 3rd generation CAR - IL-15/IL-15 sushi	- Phase I - Active	3	>18 yr	- PTCL & CTCL - Prior transplant or 4–6 standard chemotherapy	- CR = 67% (by 8 months & 15 months) - PR = 33% (by 5 months)	- CRS I–II (100%) - No neurotoxicity or severe infection - CD4^+^ T cell ablation, recovered by 3 months	NCT04162340	[59]
CD5 CAR-T	- Autologous - 2nd generation CAR	- Phase I - Active - Dose escalation - HSCT bridge	5	62–71 yr	r/r T cell NHL	- CR = 66.7% - n = 3 evaluated	- CRS I–II (60%) - Neurotoxicity II (20%) - Prolonged cytopenia (40%) - Reactivation of CMV and BK virus (20%) - No complete T-cell aplasia	NCT03081910	[60]
	- Autologous or HSCT donor - IL-15/IL-15 sushi - HSCT bridge	- Phase I - Active	1	22 yr	r/r T-LBL (CNS relapse)	- Disease remission, underwent HSCT	- CRS I - CD5^+^ T-cell aplasia, recovered by day 9 - No infection	NCT04594135	[61]
	- HSCT donor	- Phase I - Active	5	1–70 yr eligible	- r/r T-ALL - CD7-negative relapse after CD7 CAR-T cell therapy	- CR = 100% (by 1.8–4.1 months)	- CRS I–II (80%) - GvHD I (20%) - Maculopapular rash II (60%) - EBV infection IV (20%) - CD5^+^ T cell depleted - Hematological toxicity III–IV (100%)	NCT05032599	[62]
CD7 CAR-T	- Autologous - CD7 blockade with ER anchor	- Phase I - Active	5	1.9–13 yr	r/r T-ALL & T-LBL	- CR = 80% (by 1 months)	- CRS (60%), III (20%) - Hematological toxicity III–IV, recovered to II by 30 days - No neurotoxicity or infection - Reduced CD7^+^ T cell count (100%)	NCT04840875	[63]
	- Autologous - CD7 blockade with tandem CD7 nanobody	- Phase I - Completed	8	7–70 yr eligible	r/r T-ALL and T-LBL	- CR = 87.5% (by 3 months)	- CRS I–II (87.5) - No T cell hypoplasia - No neurotoxicity	NCT04004637	[23]
	- Autologous or HSCT donor - Naturally selected CD7 CAR	- Phase I - Completed	20	3–47 yr	r/r T-ALL (n = 14) & T-LBL (n = 6)	- CR = 95% (BM) by day 28 - Underwent consolidative (50%) or salvage (20%) allo-HSCT by 32-311 days	- CRS I–II (90%) - CRS III (5%) - Neurotoxicity I (10%) - Cytopenia (100%) - Sepsis (10%) - CMV reactivation (5%)	NCT04572308	[22]
	- Autologous (n = 7) or donor (n = 1) - 2nd generation CAR	- Phase I - Active	8	14–47 yr	- r/r T-LBL - 2–10 prior lines of therapies	- CR = 87.5% (BM) by day 28 - Underwent allo-HSCT (75%) by 42–56 days	- CRS I–II (87.5%) - CRS III (12.5%) - Neurotoxicity I (12.5%)	NCT04916860	[64]
	- Autologous (n = 5) or HLA-matched sibling donor (n = 5) - CD7 blocking	- Phase I - Active	10	14–70 yr	r/r T-ALL	- MRD-negative remission (n = 6, 100%)	- CRS I–II (70%) - CRS III–IV (10%) - Cytopenia IV (100%) - GvHD I–II (20%) - Multiple infections (60%) - No neurotoxicity	NCT04823091	[65]
	- HSCT donor or new donor - IntraBlock technology (CAR with ER retention of CD7)	- Phase I - Active	20	2–43 yr	- r/r T-ALL - At least 2 prior therapies - prior HSCT (n = 12)	- CR = 90%, 75% by 6.3 months - Underwent HSCT (35%)	- CRS I–II (90%) - CRS III (10%) - Cytopenia III–IV (100%) - Neurotoxicity I–II (15%) - GvHD I-II (60%) - Viral activation I–II (20%) - CD7^+^ T cell depleted	ChiCTR2000034762 Phase II: NCT04689659	[66]
CD30 CAR-T	- Autologous	- Phase I - Completed - Dose escalation	9	Child, adult, older adult eligible	- r/r HL (n = 7) & ALCL (n = 2) - 3 or more lines of prior chemotherapy	- CR=50% (n = 1) by 9 months	- No CRS - No toxicities or infections observed	NCT01316146	[67]
	- Autologous	- Phase I - Completed - Dose escalation	22	18–65 yr	- r/r PTCL, ALCL, enteropathy-associated T-cell lymphoma, extranodal NK-T-cell lymphoma	- CR = 4.8% - PR = 31.8% - SD = 57% - n = 21 evaluated	- CRS (4.5%) - Sepsis (13.6%) - Maculopapular rash (9%) - Anemia (50%) - Neutropenia (100%)	NCT03049449	[68]
	- Autologous	- Phase I - Active - Consolidation post auto-HSCT	12	16–76 yr	- r/r HL (n = 6), ALCL (n = 4), AITL (n = 1), PTCL (n = 1) - 2–3 lines of prior therapy	- PFS = 57% - OS = 77% at 1yr	- CRS I (8.3%) - No ICANS or dose-limiting toxicity (DLT) - Hematological toxicity (50%)	NCT04083495	[56]
	- Autologous - CCR4 receptor expressed	- Phase I - Active - Dose escalation	12	27–75 yr	- r/r HL (n = 10) & CTCL (n = 2) - 2–10 lines of prior therapy	- SD = 50% (n = 1)	- CRS I-II (25%) - No ICANS or DLT	NCT03602157	[55]
	- Allogeneic (Healthy donor, based on best MHC I & II match) - EBV-specific T cells	- Phase I - Active - Dose escalation	8	12–75 yr eligible	- r/r CD30^+^ lymphoma - 3–5 lines of prior therapy	- CR = 28.5% (n = 2) - PR = 42.9% (n = 3) - n = 7 evaluated	- No CRS, DLT, or GvHD	NCT04288726	[58]
TRBC1 CAR-T	- Autologous	- Phase I/II - Active - Dose escalation and expansion	10	34–63 yr	- r/r PTCL NOS (n = 5), AITL (n = 4), ALCL (n = 1) - 1–5 lines of prior therapy	- Complete metabolic response = 55.5% (n = 5) - PR = 11.1% (n = 1) - n = 9 evaluated at 1 month	- Cytopenia (anemia & neutropenia) - CRS I–II (30%) - CRS III (10%) - No neurotoxicity/ICANS or DLT - No grade III or higher infections	NCT03590574	[69]

**Table 4:** Advantages and limitations of conventional T cells, NK cells, γδ T cells, and DNT cells as a vehicle for CAR.

	CAR- Conventional T/CD8^+^	CAR-NK	CAR-γδ T	CAR-DNT
Risk of GvHD	High due to alloreactive TCRs - Studies investigating genetic modifications (e.g., TCR KO) or non-alloreactive T cells (e.g., virus-specific)	Low - Protective against GvHD activity by targeting recipient APCs	Low	Low - GvHD suppressive activity
Risk of Immune Rejection	High due to MHC mismatch - Studies investigating genetic modifications (e.g., MHC I/II KO) or inhibition of T cell & NK cell cytotoxicity (e.g., certain MHC I alleles)	Present - Require lymphodepletion to suppress T cell activity to minimize NK graft rejection (especially when IL-15 supplementation is used) - Studies investigating genetic modifications (multiple KOs/knock-ins)	Unclear	Low - Resistant to rejection
Risk of Fratricide	Present - Studies investigating surface antigen KOs - Antigens restricted to specific T cell subsets	None for T-lineage specific antigens	Depends on target antigen - No fratricide for TCR αβ	Depends on target antigen - No fratricide for CD4 or CD8
Lifespan/ Persistence	Longest persistence - Detectable for 6 months to years after therapy	Shorter - Detectable for only 3 weeks; lacks long-term antitumor efficacy - Requires multiple doses, increasing risk of rejection - Studies investigating memory-like NK	Shorter	Shorter
Antitumor Cytotoxicity	MHC-dependent - No endogenous killing ability with TCR KO	MHC independent - NK cell receptors - ADCC, potential use in combination with antibody treatment	MHC independent - TCRγδ - NK cell receptors - ADCC	MHC independent - TCRγδ - TCRαβ - NK cell receptors
CAR Construct Suitability	Superior cytotoxicity - CAR originally designed for T cells	Inferior cytotoxicity compared to conventional T cells -NK signaling might affect performance (studies investigating NK-specific CAR constructs)	Comparable with conventional CAR-T	Comparable with conventional CAR-T
Toxicities/ Side Effects	- Cytokine release syndrome (CRS) - Studies investigating use of safety switch to prevent T-cell aplasia or severe adverse events	- Reduced risk of CRS due to limited cytokine secretion profile - Studies suggesting limited persistence can reduce risk of T-cell aplasia	Limited CRS	Unclear
Cost	High due to necessary modifications	Depends on source of cells	Low	Low
Sources	Readily available - Peripheral blood (PB) - Umbilical cord blood	- 10% PB, mature phenotype, harder to expand and standardize product - Umbilical cord blood, immature phenotype - NK-92 cell line commonly used, needs to be irradiated before use, reduced proliferative capacity, derived from lymphoma - iPSC	1–10% of PB T cells	3–5% of PB T cells
Culture and Expansion	Can be expanded to therapeutic numbers	- Poorer expansion (from PB) than conventional T cells - May involve use of feeder cells (risk of contamination, more complicated/costly)	Can be expanded to therapeutic numbers	Can be expanded to therapeutic numbers
Transduction Efficiency	High	Lower - May require multiple transductions or cell sorting	Comparable with conventional CAR-T	Comparable with conventional CAR-T
Cryopreservation	Can be cryopreserved	More sensitive to freezing/thawing than conventional T cells	Sensitive to freezing/thawing	Maintain viability and antitumor functions
Dependence on Cytokine Support		Yes	Yes	Yes

**Table 5:** Clinical trials of NK cell therapy for T-cell malignancies.

Treatment Approach	Disease	Other Therapy	Title	Study Stage	Phase	Clinical Trial Identifier
Expanded haploidentical NK cells	r/r T-ALL	Post HSCT Concurrent chemotherapy	Pilot Study of Expanded, Donor Natural Killer Cell Infusions for Refractory Non-B Lineage Hematologic Malignancies and Solid Tumors	Completed	Phase I	NCT00640796
Donor NK cells	Recurrent or Stage III/IV adult T cell leukemia/lymphoma	Post HSCT	Donor NK Cell Infusion for Progression/Recurrence of Underlying Malignant Disorders After HLA-haploidentical HCT–a Phase 1-2 Study	Completed	Phase I/II	NCT00823524
Third party NK cells	Recurrent or r/r adult T cell leukemia/lymphoma Recurrent or r/r primary cutaneous T cell NHL	Monoclonal antibody (Mogamulizumab)	A Pilot Phase I Trial of IL-21 Expanded, Off the Shelf, Third-Party Natural Killer (NK) Cells in Combination With Mogamulizumab in Patients With Cutaneous T-Cell Lymphomas or Adult T-Cell Leukemia/Lymphomas	Recruiting	Phase I	NCT04848064
Expanded, activated haploidentical NK cells	r/r/ T cell lymphoblastic leukemia and lymphoma	Concurrent Salvage Chemotherapy	Salvage Therapy With Chemotherapy and Natural Killer Cells in Relapsed/Refractory Paediatric T Cell Lymphoblastic Leukaemia and Lymphoma (HNJ-NKAES-2012)	Terminated due to low recruitment		NCT01944982
Expanded, activated haploidentical NK cells	T-ALL	Concurrent Chemotherapy	Pilot Study of Expanded, Activated Haploidentical Natural Killer Cell Infusions for Non-B Lineage Acute Leukaemia and Myelodysplastic Syndrome	Unknown	Phase I	NCT02123836

**Table 6:** Clinical trials of γδ T cell therapy for T-cell malignancies.

Treatment Approach	Disease	Other Therapy	Title	Study Stage	Phase	Clinical Trial Identifier
Ex vivo expanded allogeneic γδ T cells	r/r PTCL	None	The Safety and Efficacy Assessment of Ex vivo Expanded Allogeneic γδT Cells Immunotherapy in Patients With Relapsed or Refractory Non-Hodgkin’s Lymphoma (NHL) and Peripheral T Cell Lymphomas (PTCL)	Recruiting	Early Phase I	NCT04696705
Ex vivo expanded γδ T cells	ALL	Post HSCT	Phase I Study of Ex Vivo Expanded/Activated Gamma Delta T-cell Infusion Following Haploidentical Hematopoietic Stem Cell Transplantation and Post-transplant Cyclophosphamide	Recruiting	Phase I	NCT03533816
Ex vivo expanded γδ T cells	ALL	Post HSCT	Safety and Efficiency of γδ T Cell Against Hematological Malignancies After Allo-HSCT. A Dose Escalation, Open-label, Phase 1/2 Study.	Recruiting	Phase I/II	NCT04764513
Ex vivo expanded γδ T cells	ALL	Post HSCT Concurrent chemotherapy CD45RA-depleted donor memory T cells	TCRαβ-depleted Progenitor Cell Graft With Additional Memory T-cell DLI, Plus Selected Use of Blinatumomab, in Naive T-cell Depleted Haploidentical Donor Hematopoietc Cell Transplantation for Hematologic Malignancies	Recruiting	Phase II	NCT03849651
